# Adenosine induced pulmonary vasodilation is blocked by active cigarette smoking, an evaluation by pulmonary transit time with first pass perfusion MRI

**DOI:** 10.1186/1532-429X-11-S1-O41

**Published:** 2009-01-28

**Authors:** Jie Jane Cao, Sophie Wang, Marguerite Roth, William Schapiro, Yi Wang, Nathaniel Reichek

**Affiliations:** St Francis Hospital, Roslyn, NY USA

**Keywords:** Pulmonary Vasculature, Purine Nucleoside, Adenosine Infusion, Pulmonary Vasodilation, Past Smoker

## Introduction

Pulmonary transit time (PTT) measures the time that blood travels through the pulmonary vasculature. PTT correlates well with pulmonary vascular resistance. It is prolonged in patients with pulmonary hypertension and congestive heart failure. Similar to systemic and coronary vasculature, the pulmonary arteries dilate in response to purine nucleoside adenosine which has a direct endothelium independent effect on the A2b receptor in vascular smooth muscle. Despite the detrimental effect of cigarette smoking on endothelium dependent systemic vascular function, little is known of its effect on endothelium independent function of the pulmonary vasculature.

## Purpose

The objective of this study is to develop a non-invasive strategy using first pass perfusion MRI to measure PTT and to test the hypothesis that cigarette smoking inhibits endothelium independent pulmonary vasodilation.

## Methods

The study included 63 prospectively recruited subjects (65% women) without significant pulmonary disease. All subjects underwent first pass perfusion cardiac MRI in a 1.5 Tesla Siemens scanner. Three long axis planes were acquired per heartbeat over 50 heartbeats using a partial Fourier saturation-recovery steady state free precession sequence during a breath hold. Dynamic perfusion imaging was first performed during adenosine infusion at 140 μg/kg/min with gadolinium concentration at 0.05 mmol/kg. Following a 20 minute washout period dynamic imaging was repeated without adenosine using the same parameters. Images were analyzed in commercial software (Argus, Siemens, Germany). PTT was measured between the times when signal intensity reached a peak in main pulmonary artery and in the left atrium.

## Results

The mean age of the study cohort was 55.9 ± 13.1 years. The prevalence of never, past and current smoking was 42.4%, 49.2% and 8.5%, respectively. Compared to rest PTT (5.98 ± 1.33 s) there was a 25.4% reduction (p < 0.001) in PTT during adenosine infusion (4.46 ± 0.94 s). When the analysis was stratified by smoking status significant PTT reduction was seen in never smokers (25.5%, p < 0.001) and in past smokers (28.5%, p < 0.001) but not in the current smokers (8.8%, p = 0.433). However, there was significant correlation between R-R interval and PTT with Spearman correlation coefficient 0.640 (p < 0.001) for rest PTT and 0.551, (p < 0.001) for stress PTT. In view of a 27.5% heart rate increase (p < 0.001) the analysis was repeated using PTT values normalized to RR interval. In contrast to absolute PTT measurements the normalized values demonstrated minimal change between rest and stress PTT in never smokers (0.06%, p = 0.989) and in past smokers (-1.55%, p = 0.651). In contrast, there was an 18.7% increase (p = 0.029) in normalized stress PTT among current smokers.

## Conclusion

Adenosine infusion was associated with significant PTT shortening in never and past smokers. Shortened PTT likely represented reduced pulmonary resistance in response to the endothelium independent vasodilatory effect of adenosine. However this response appeared to be blocked by active cigarette smoking. In the absence of active cigarette smoking PTT normalized to R-R interval remained constant at rest and during stress, suggesting an intact vasoreactivity to accommodate increased cardiac output during adenosine infusion. Conversely this value was significantly prolonged during adenosine infusion among current smokers, implying impaired vasoreactivity likely due to the detrimental effect of cigarette smoking, which resulted in delay of pulmonary blood transit in the presence of increased blood volume. Our findings suggest that CMR adenosine stress testing may be an important modality to evaluate pulmonary arterial function.

